# Influence of Demographic Factors on Sheepmeat Sensory Scores of American, Australian and Chinese Consumers

**DOI:** 10.3390/foods9040529

**Published:** 2020-04-22

**Authors:** Rachel A. O’Reilly, Liselotte Pannier, Graham E. Gardner, Andrea J. Garmyn, Hailing Luo, Qingxiang Meng, Markus F. Miller, David W. Pethick

**Affiliations:** 1 Australian Cooperative Centre for Sheep Industry Innovation, Armidale, New South Wales 2351, Australia; L.Pannier@murdoch.edu.au (L.P.); G.Gardner@murdoch.edu.au (G.E.G.); D.Pethick@murdoch.edu.au (D.W.P.); 2 College of Science, Health, Engineering and Education, Murdoch University, Perth, Western Australia 6150, Australia; 3 Department of Animal and Food Sciences, Texas Tech University, Lubbock, TX 79409, USA; andrea.garmyn@ttu.edu (A.J.G.); markus.miller@ttu.edu (M.F.M.); 4 State Key Laboratory of Animal Nutrition, College of Animal Science and Technology, China Agricultural University, Beijing 100083, China; hailingluo_cau@126.com (H.L.); qxmeng@cau.edu.cn (Q.M.)

**Keywords:** consumer, demographic, sensory, lamb, yearling, longissimus, semimembranosus, cross-cultural

## Abstract

Along with animal production factors, it is important to understand whether demographic factors influence untrained consumer perceptions of eating quality. This study examined the impact of demographic factors and sheepmeat consumption preferences on eating quality scores of American, Australian and Chinese untrained consumers. M. *longissimus lumborum* (LL) and m. *semimembranosus* (SM) were grilled according to sheep Meat Standards Australia protocols and evaluated by 2160 consumers for tenderness, juiciness, flavour and overall liking. Linear mixed effects models were used to analyse the impact of demographic factors and sheepmeat consumption habits on eating quality scores. Consumer age, gender, number of adults in a household and income had the strongest effect on sensory scores (*P* ≤ 0.05), although, the impact was often different across countries. Frequency of lamb consumption had an impact on sensory scores of American, Australian and Chinese consumers but larger sample sizes in some underrepresented subclasses for Australian and Chinese consumers are needed. Results suggest it is important to balance sensory panels for demographic factors of age, gender, number of adults and income to ensure sensory preferences are accurately represented for these particular populations.

## 1. Introduction

As in beef, the development of a sheep Meat Standards Australia (MSA) grading system is underpinned by sensory evaluations of vast numbers of untrained consumers [1,2,3]. The MSA prediction model forecasts consumer eating quality of a final cooked product, and is based on consumer scoring of a variety of muscles using descriptors of tenderness, juiciness, liking of flavour and overall liking [1,4]. This prediction model is successfully implemented for beef and is being developed for sheep [3]. While the merit of using untrained consumers is that they are by definition unbiased, some potential drawbacks can include high variance of consumer scores, and the risk of sampling from discreet sectors of the community not representative of an entire population. Previously, O’Reilly et al. [5] demonstrated minor differences in sensory scores of grilled Australian sheepmeat between American, Australian and Chinese consumers, with a consistent response across consumer groups to production factors of muscle type, animal age and sire type. However, the influence of demographic factors and meat consumption habits on sensory scores particular to these culturally unique groups remained unexplored. Lamb consumption rates in the USA are markedly lower than for Australian consumers, 0.6 kg compared to 8.6 kg per person annually [6], therefore many American consumers are unfamiliar with this protein. Similarly, Chinese consumers would largely be unaccustomed to Western style grilled lamb with the top three sheepmeat cooking styles in China being stewing, roasting or hotpot [7,8].

Previous research has shown that consumer demographic factors and meat consumption habits have some impact on eating quality scores in sheep [9] and beef [10], however the magnitude of these effects are often low, inconsistent across eating quality traits, and differ across countries. Australian, French, Northern Irish and Polish consumers with a higher appreciation of red meat in their diet tend to score lamb and beef more favourably than those who classify themselves as indifferent to red meat [9,10]. This was not observed for Irish or Korean consumers rating grilled beef samples [10,11]. A preferred higher degree of cooking doneness has also been shown to positively influence sensory scores compared to those who prefer medium doneness for Australian, Northern Irish and Irish consumers, however no effect was observed in French or Korean consumers, with Polish consumers demonstrating the opposite effect [9,10,11].

Similar to meat consumption habits, demographic factors of gender, consumer age, occupation, income level and household size have an inconsistent impact on eating quality scores across the literature [9,10,11,12]. For example, in European comparisons, Northern Irish, Irish and Polish males scored grilled beef up to two units higher than females [10]. Similarly, Kubberød et al. [13] demonstrated higher scores for Norwegian males than females when assessing lamb and beef. Both studies attributed this effect to males, placing a higher degree of importance of meat in their diet compared to females [10,13]. In contrast, Thompson et al. [9] found Australian females to score lamb up to two units higher than males. While Huffman et al. [12] and Hwang et al. [11] found no effect of gender on sensory scores for American, Korean and Australian consumers scoring beef grilled and barbequed Korean style. Older consumers within Australia have demonstrated higher sensory scores than younger consumers [9,14], while an inconsistent response to age was observed in the USA [12]. On the other hand, a small negative relationship in eating quality scores was observed with increasing consumer age for tenderness in France and Poland, and for juiciness in Ireland, Northern Ireland and Poland [10]. Huffman et al. [12] found as income increased, sensory scores decreased, with the assumption that participants on higher incomes have greater expectations of the products they consume. In addition, a greater number of adults in the household has also yielded slightly higher eating quality scores in some studies [9,10].

Largely, these studies imply that for sensory evaluations of beef and sheepmeat using untrained consumers, demographic factors pose a relatively unimportant source of bias on sensory scores [9,10,11,12,13]. However, to accurately assess American and Chinese consumer perceptions of sheepmeat, it is important to assess whether their demographic factors play a role in sensory scoring as this has not been reported before. In addition, lambs have demonstrated greater palatability than yearlings and mutton for Australian, American and Chinese consumers [5,15,16]. Hence, it is important to investigate whether demographic factors have an effect on animal age preferences. Therefore, this study examined the effect of consumer demographic factors on eating quality scores of American, Australian and Chinese consumers testing grilled Australian lamb and yearling m. *longissimus lumborum* (LL) and m. *semimembranosus* (SM) muscles. We hypothesised that demographic factors would have a small and inconsistent effect on eating quality scores across countries, and that higher appreciators of meat and those that prefer their meat medium-well done will score more favourably. In addition, given similarities between Korean and Chinese culinary habits, we hypothesised that Chinese eating quality scores would be less responsive to changes in meat consumption preferences compared to American and Australian counterparts.

## 2. Materials and Methods

### 2.1. Animal and Muscle Collection

Carcasses used in this study were described in detail by O’Reilly et al. [5]. In brief, a total of 164 lambs (no erupted permanent incisors; average age 368 days) and 168 yearlings (2–4 erupted permanent incisors; average age 726 days) were included in this experiment. Lambs (females and wethers) were the progeny of Maternal (Border Leicester, and Dohne Merino), Merino (Merino, and Poll Merino), and Terminal (Poll Dorset, Suffolk, Texel and White Suffolk) sires, whereas yearlings (wethers) were the progeny of Merino (Merino, and Poll Merino) sires only. Animals were sourced from the Meat and Livestock Australia genetic resource flock at Kirby (NSW, Australia) [17]. Animals were commercially slaughtered at an abattoir licensed for international export to China and the USA. Medium voltage electrical stimulation [18] was applied to all carcasses, before being trimmed according to AUS-MEAT specifications [19], and chilled for 24 h at 3–4 °C prior to sampling. Left and right LL (AUS-MEAT 5150) and SM (AUS-MEAT 5077) muscles were collected from each carcass at 24 h postmortem, vacuum packed and aged for ten days at 2–3 °C prior to frozen transport to China and the USA or retained within Australia. All samples were transported according to commercial processing plant specifications with temperatures maintained below minus 10 °C. A total of 648 LL, 648 SM muscles were collected with every carcass represented in two of three countries at any one time through allocation of muscles collected.

### 2.2. Sample Preparation and Sensory Testing

Consumer assessment of the sheepmeat was performed according to MSA testing protocols detailed by Thompson et al. [2] and Watson et al. [20], with minor updates described by O’Reilly et al. [5]. In brief, 24 h prior to each session, muscle cuts were thawed at 2–5 °C, each muscle was then sliced into five 15 mm thick steaks (approximately 5 × 5 × 1.5 cm), and allocated to consumers using a 6 × 6 Latin square design. Steaks were grilled on a Silex griller (electric S-Tronic Single Grill S161GR, Hamburg, Germany) with top and bottom grill plates set to 180 °C and 195 °C to obtain a medium doneness (2.25 min; 65 °C internal temperature). Steaks were rested (1.5 min) before being halved and served to consumers seated in individual booths, resulting in 10 samples per muscle being served and tasted. The same model of grill was used in Australia, China and the USA.

A total of 2160 untrained consumers were recruited across Australia, China and the USA to participate in one of the 12 tasting sessions conducted per country (*n* = 720 consumers per country, 60 per session). Each consumer scored six samples (3 LL and 3 SM) for tenderness, juiciness, liking of flavour, and overall liking on a hedonic scale line of 0–100. Scale lines were anchored with ‘not’ and ‘very’ preceding the eating quality trait for tenderness and juiciness, and ‘dislike extremely’ and ‘like extremely’ for liking of flavour and overall liking. In Australia and the USA, participants largely consisted of community organisations and clubs recruited through a market research company and Texas Tech University. While in China, participants were recruited though China Agricultural University and localised to the community surrounding the university. Tasting sessions in Australia were conducted in the outer Melbourne suburbs of Victoria, at the China Agricultural University in Beijing, China and across ten American states: California, Colorado, Florida, Kansas, Ohio, Oklahoma, Pennsylvania, South Carolina, Texas and Utah.

### 2.3. Consumer Demographics

In addition to scoring sheepmeat samples, consumers were asked to fill in a questionnaire on their demographic details and meat consumption preferences. Recruitment requested participants aged between 18 and 70 years old, preferred to be consumers of sheepmeat at least once per fortnight and have a preference for their meat to be cooked to a medium level of doneness. The questions asked were:

1. Consumer Age group, based on 6 categories: *(a)* <20, *(b)* 20–25, *(c)* 26–30, *(d)* 31–39, *(e)* 40–60, *(f)* 61–70;
2. Gender, based on two categories: *(a)* male, *(b)* female;
3. Number of adults in the household, based on 5 categories: 1, 2, 3, 4, ≥5;
4. Number of children in the household, based on 6 categories: 0, 1, 2, 3, 4, ≥5;
5. Income per annum, based on 5–8 categories depending on country: Australia in AUD: *(a)* <$25,000, *(b)* $25,000–$50,000, *(c)* $50,001–$75,000, *(d)* $75,001–$100,000, *(e)* $100,001–$125,000, *(f)* $125,001–$150,000, *(g)* >$150,000 *(h)* Prefer not to say; China in yuan: *(a)* ≤¥24,000, *(b)* ¥24,000–¥36,000, *(c)* ¥36,001–¥60,000, *(d)* ¥60,001–¥96,000, *(e)* ¥96,001–¥120,000, *(f)* >¥120,000, *(g)* Prefer not to say; and the USA in USD: *(a)* <$20,000, *(b)* $20,000–$50,000, *(c)* $50,001–$75,000, *(d)* $75,001–$100,000, *(e)* >$100,000.
6. Occupation, based on 8–11 categories depending on country: for Australia and China *(a)* manager, *(b)* professionals, *(c)* technician and trade workers, *(d)* community and personal service, *(e)* administration, *(f)* sales and service, *(g)* machinery operators and drivers, *(h)* labourers, *(i)* home duties, *(j)* student, *(k)* other; and for the USA *(a)* professionals, *(b)* tradesperson, *(c)* administration, *(d)* sales and service, *(e)* labourers, *(f)* home duties, *(g)* student, *(h)* other;
7. Consumption frequency, based on seven categories: for Australia and China *(a)* daily, *(b)* 4–5 times per week, *(c)* 2–3 times per week, *(d)* weekly, *(e)* fortnightly, *(f)* monthly, *(g)* never eat lamb; and for the USA *(a)* daily, *(b)* weekly, *(c)* fortnightly, *(d)* monthly, *(e)* every other month, *(f)* 2–3 times per year, *(g)* never eat lamb;
8. Preference for lamb, based on four categories: *(a)* Appreciator: ‘I enjoy lamb. It’s an important part of my diet’, *(b)* Lamb is important: ‘I like lamb well enough. It’s a regular part of my diet’, *(c)* Indifferent: ’I do eat some lamb although, truthfully it wouldn’t worry me if I didn’t’, *(d)* Rarely/never eat: ‘I rarely/never eat lamb’;
9. Preferred degree of cooking doneness, based on 6 categories: *(a)* blue, *(b)* rare, *(c)* medium/rare, *(d)* medium, *(e)* medium/well done, *(f)* well done.

### 2.4. Statistical Analysis

The effect of demographic factors on eating quality scores (tenderness, juiciness, liking of flavour and overall liking) were examined using linear mixed effects models in SAS (SAS Version 9.1, SAS Institute, Cary, NC, USA). All ten consumer responses for each muscle were included in the analysis to account for the consumer variation. Initially, base models were established for tenderness, juiciness, liking of flavour and overall liking. These included fixed effects for country (Australia, China, USA), muscle (LL, SM), sex within animal age class (female lamb, wether lamb and wether yearling), sire type within animal age class (Maternal lamb, Merino lamb, Terminal lamb and Merino yearling), and kill group (animals were slaughtered in two separate groups), which were only retained if significant (*P* < 0.05). These base models have previously been reported in O’Reilly et al. [5] and enabled the models to account for any imbalance in animal type inadvertently occurring across demographic factor categories. Demographic factors were then included together in each of the base models as fixed effects. These included consumer age group, gender, income, occupation, adults in household, children in household, frequency of lamb consumption, preferred cooking doneness and importance of lamb in diet. Income level, occupation and frequency of lamb consumption were fitted within-country because their category options were defined differently between countries. In addition, preferred degree of doneness was also fitted within country, in this case because there were missing subclasses of ‘blue’ for the USA and ‘blue and rare’ for China. All demographic factors were interacted with muscle and with country, except those nested within country (income level, occupation, consumption frequency and preferred doneness). Non-significant terms (*P* > 0.05) were removed in a stepwise manner. Random terms included consumer identification within tasting session by country, and animal identification within sire identification. The Satterthwaite function was used in all models to approximate the degrees of freedom.

## 3. Results

### 3.1. Demographic Factor Distribution

Table 1 shows the demographic distribution of consumers recruited within Australia, China and the USA. Of the 2160 participants that attended tasting sessions, 2117 were included in the analysis as they had data for all demographic categories. Consumer age group varied by country with Australian consumers generally being older with more than half over 40 years of age, while American and Chinese consumers were largely aged between 20 and 25 years old. Gender was consistent for Australia and the USA with 10% more males than females, while China had 20% more females than males.

The majority of American and Australian consumers came from households of one to three adults, while Chinese participants mainly came from households of three or above. All three countries leant towards less children (≤2), but this was more prominent for Chinese and American consumers. Australian and American consumers were evenly distributed across income brackets while Chinese consumers were largely within the lowest income category. Australians classified themselves primarily as managers, professionals and trade workers, Americans as administrators and homemakers, and Chinese as professionals and students. Lamb consumption habits were varied across the three countries. Most Australian and Chinese consumers eat lamb weekly to monthly, with a tendency towards higher and lower consumptions rates respectively. American consumers largely ate little to no lamb with over 70% of participants in the ‘2–3 times per year’ to ‘never eat’ categories. Australians were the highest appreciators of lamb, with the majority indicating lamb is important in their diet. Chinese consumers were largely indifferent to inclusion of lamb in their diet, and the majority of American consumers were either indifferent to lamb or never eat it. Preferred degree of cooking doneness was similar for American and Australian consumers with close to 90% of participants evenly distributed across medium/rare to medium/well done categories. In contrast, almost all Chinese consumers preferred their meat cooked from medium to well done.

### 3.2. The Impact of Demographic Factors on Sensory Scores

Table 2 presents significance levels of demographic factors included in base models for tenderness, juiciness, liking of flavour and overall liking. Consumer age group, number of adults in the household, income level and frequency of lamb consumption reached significance most often across the four sensory traits while occupation, consumer appreciation of lamb and preferred degree of doneness were not significant for any sensory trait. Consumer gender and number of children in a household had a limited effect on sensory traits (Table 2).

#### 3.2.1. Consumer Age Group

On average, across the three countries and both muscles, scores for tenderness, flavour and overall liking scores varied between consumer age group (*P* < 0.05; Table 2). Consumers ≥40 years old scored tenderness 3.0 to 6.3 units higher than younger counterparts, while 26 to 30 year olds scored flavour and overall liking 3.0 to 5.7 units lower than both younger and older age groups.

The age group effect varied between countries and muscle types for tenderness, juiciness, flavour and overall liking (*P* < 0.05; Table 3). As a general trend, younger Australian consumers tended to score eating quality highest, while the reverse was observed for Chinese and American consumers with older participants scoring more favourably. In Australia, younger consumers tended to score tenderness and juiciness higher. Consumers ≤25 years old scored 5.0 to 9.1 tenderness units higher than 26 to 60 year olds. For juiciness, this trend remained for the SM muscle, with consumers ≤25 years old scoring samples 5.9 to 10.6 units higher than those aged 26 to 60 years old. However, for the LL muscle, juiciness scores differed across the older consumer groups (*P* < 0.05; Table 3).

In contrast, Chinese consumers ≥40 years old and American consumers ≥31 years old scored tenderness and juiciness 3.6 to 8.6 units higher than most younger age groups. Though, for Chinese consumers, improved scoring of juiciness (4.2 units) was only observed in the LL for 40 to 60 year olds compared to 20 to 25 years, whereas in the SM, the effect extended to those above 60 years compared to most age group categories under 40 years old differing by about 4.4–7.8 units. American consumers ≥40 years old scored LL samples 5.2 to 6.0 juiciness units greater than 20 to 30 year olds, while for the SM, 26 to 30 year olds scored juiciness 4.8 to 8.7 units lower than almost every other age group (*P* < 0.05; Table 3).

Aligning with tenderness and juiciness, when averaged across both muscles, younger Australian consumers scored flavour (4.7–6.1 units) and overall liking (5.1–6.3 units) more favourably than older consumers. Notably, those aged 26 to 30 years old scored flavour and overall liking on average 4.7 to 10.2 units lower than all other age groups. This trend was evident in the SM muscle for which flavour and overall liking scores were highest amongst those ≤ 25 years old, with increases of 6.2 to 14.5 units compared to categories upwards of 26 years old. However, this trend differed within the LL, as Australian consumers over 60 scored flavour higher than those aged 26 to 30 and 40 to 60 years old (6.2 and 4.8 units). Similarly, overall liking scores in the LL were significantly greater for those over 60 and under 20 compared to the 26 to 30 years-old category (6.6 and 6.2 units).

Older Chinese consumers scored flavour (≥40 years) and overall liking (≥60 years) 5.2 to 7.5 units higher than younger consumers (≤25 years old). There was no impact of age group seen for LL samples, however, in the SM muscle, older Chinese consumers (generally ≥40 years old) scored flavour and overall liking more favourably than those in categories ≤25 years old (4.7–11.7 units).

Similar to some Australian participants, American consumers aged 20 to 30 years old scored flavour and overall liking lower than younger and most older age groups by 3.2 to 7.3 units (*P* < 0.05; Table 3). This effect was seen in the LL samples with consumers aged 20 to 25 years old and 20 to 30 years old scoring flavour (4.4 units) and overall liking (~5 units) lower than consumers aged 40 to 60 years old. Similarly, for the SM muscle, 20 to 30 year olds scored significantly lower for flavour and overall liking than all other age groups (4.6–9.0 units) (*P* < 0.05; Table 3).

Country differences were also observed within consumer age groups (*P* < 0.05; Table 3). Across muscle types, American tenderness scores were greater than Chinese consumers for the age groups under 20, and 31–39 years old (~10.1 units), and Australian consumers 40–60 years old (6.8 units). In addition, Australian tenderness scores were higher than Chinese in the under 20-year-old category (12.7 units). Australian consumers scored juiciness higher than Chinese consumers ≤25 years old (10 units), and 31 to 39 years old (7.7 units) and American consumers 20 to 25 years old (5.4 units). American consumers within the 31 to 39 years-old category also scored juiciness 7.2 units higher than Chinese consumers. No differences between countries was observed for tenderness in age categories 20 to 30 years old and above 60 years old, and for juiciness in groups 26 to 30 and above 40 years old. Country differences across age groups were generally consistent for flavour and overall liking with Australian and American consumer scores greater than Chinese scores for those under 20 years old (7.2 to 12.4 units). Within consumer group 20 to 25 years old, Australian flavour and overall liking scores were greater than American and Chinese consumers by 6.1–8.8 units. There were no country differences for flavour and overall liking within age groups above 26 years old (*P* < 0.05; Table 2).

#### 3.2.2. Number of Adults and Children in a Household

Number of adults in a household when averaged across all countries and muscle types had an impact on tenderness, juiciness, flavour and overall liking scores (*P* < 0.05; Table 2; individual data not shown). In general, households with more adults present scored higher eating quality scores than those containing less adults. Households with 3 adults scored tenderness, and juiciness higher than households with 1 and 2 adults (3.0 units; *P* < 0.05), and 1, 2 and 5 adults (3.0 to 3.3 units; *P* < 0.05) respectively. Similarly, flavour and overall liking was also scored highest by households of 3 adults compared to 2 and 4 adult households (1.8 to 3.2 units; *P* < 0.05). These trends differed across country and muscle for tenderness and juiciness (*P* < 0.05; Table 2).

Within Australia, LL samples scored 6.6 tenderness units higher in households of 4 adults compared to 5, while households of 3 and 5 adults scored SM samples 6.2 to 7.2 units higher than 1 and 2 adults. Within China, households of 5 adults scored LL tenderness about 4.9 units higher than 1 and 4 adults, and within the USA households with 1 and 3 adults scored LL tenderness 4.7 and 5 units higher than 2 adults. Number of adults within a household did not have an impact on tenderness scoring of the SM for American and Chinese consumers. For juiciness, on average across both muscles, Australian consumers with households of 3 and 4 adults scored 3.8 to 5.8 units higher than all other categories. Within China juiciness scores did not differ based on number of adults in a household. American consumers of households with 1 and 3 adults scored juiciness 4.6 to 6.5 units higher than households of 2 and 5 adults.

Comparing within adult categories (Table 2; individual data not shown), American tenderness LL scores were greater than Australian for 1 and 5 adults (7.4 and 10.4 units; *P* < 0.05) and Chinese in 1, 3 and 4 adults in a household (9.3 to 12.4 units; *P* < 0.05). SM scores largely did not differ by country. Within adult categories, Australian consumer juiciness scores were greater than Chinese for 2, 3 and 4 adults (4.7 to 5.8; *P* < 0.05) and American for 4 adults in a household. American juiciness scores were also higher than Chinese for 1 and 3 adults in a household (9.6 and 4.7 units). There was no difference in countries for 5 adults in a household.

Number of children within a household only had an impact on tenderness scores (*P* < 0.05; Table 2; individual data not shown). American consumers reported no tenderness differences in LL samples across the number of children in a household, whereas for the SM households with 5 children scored tenderness 12.9 to 16.1 units higher than those with 0 to 3 children (*P* < 0.05). Within Australia and China, the number of children within a household had no detectable impact on tenderness scores. Differences across the three countries were similar to those found for other demographic traits. For households with 0 and 1 children, Australian and American consumers scored tenderness 7.0 to 10.3 units higher than China for the LL and SM. For households of 2 and 3 children, Americans scored the LL higher than Chinese consumers (9.5 and 12.3; *P* < 0.05), and for households of 5 children, Americans score the SM 13.9 units greater than Australians (*P* < 0.05). No country difference was reported for SM samples in 3 children, LL and SM samples in 4 children and LL samples in households of 5 children.

#### 3.2.3. Consumer Gender

Consumer gender had a significant impact on flavour and overall liking, but only for American consumers (*P* < 0.05; Table 2). American males scored flavour and overall liking 3.6 and 3.5 units higher than females (*P* < 0.05; individual data not shown) However, country differences were detected with American and Australian males scoring flavour and overall liking higher than Chinese males (4.0–6.4 units; *P* < 0.05; individual data not shown), while female scores across the three countries were not significantly different.

#### 3.2.4. Consumer Income

Within each country, consumer income bracket had a significant impact on sensory scores of tenderness, flavour and overall liking (*P* < 0.05; Table 2 and Table 4). This effect varied within each country, though a cross-country comparison could not be made as the local currency was used in questionnaires. Overall, income had the greatest influence on sensory scores for Australian consumers of low income, and middle-income Chinese consumers.

Australian consumers within lower income brackets tended to score tenderness, flavour and overall liking higher than those within higher income brackets with the exception of those within the >$150,000 category (Table 4). For tenderness, on average, consumers with income <$100,000 scored 3.9 to 9.3 units higher than those in higher income brackets. For flavour, the two income brackets ranging from $25,000 to $75,000 demonstrated scores 3.7 to 6.3 units higher compared to categories $75,000 to $120,000. Similarly, average overall liking scores were greatest for lower income earners (<$75,000) with increases ranging from 4.3 to 8.1 units compared to higher income categories. This income difference also varied slightly between muscle types for flavour and overall liking (*P* < 0.05; Table 2; individual data not shown) but typically lower income earners scored flavour and overall liking more favourably than higher income earners both in LL and SM muscle (*P* < 0.05; Table 2 and Table 4; individual data not shown).

Within China, there was no consistent trend observed for income bracket. Tenderness only differed amongst those who preferred not to divulge their income and earners within the ¥60,001–¥96,000 category. For flavour and overall liking, on average, consumers within the income bracket ¥36,001 to ¥60,000 scored 4.7 to 5.3 units lower than the income brackets immediately above and below. This varied between the muscles, flavour LL scores increased with higher income up to ¥96,000, while improvements in scoring were limited to bracket ¥24,000 to ¥36,000 for the SM. Similarly, greater overall liking scores of the LL were restricted to ¥24,000 to ¥36,000 (*P* < 0.05; Table 2 and Table 4; individual data not shown) with no detectable difference for the SM.

Similar to Chinese consumers, there was no consistent impact of income on sensory scores for American consumers. Participants within income bracket US$75,001 to $100,000 scored tenderness on average 3.5 units higher than income brackets immediately above and below. Flavour scores were on average 3.8 to 4.4 units higher amongst the income brackets <US$50,000 and US$75,001 to $100,000, following the same trend for LL and SM muscles (3.8 to 4.8 units higher). Overall liking scores were on average, 3.5 to 4.9 units higher in earners <US$50,000 per annum, again with LL and SM following the same trend (*P* < 0.05; Table 2 and Table 4; individual data not shown).

#### 3.2.5. Meat Consumption Habits

Of all meat consumption habits analysed, only frequency of consumption had a significant impact on eating quality scores. This was observed for tenderness, juiciness, flavour and overall liking and varied across muscles (*P* < 0.05; Table 2 and Table 5). The general trend was similar across countries with higher consumption frequency having a positive influence on sensory scores, however statistical comparisons between countries was not possible given different scales were used. Typically, more frequent lamb consumption habits increased eating quality scores, however for LL samples, Australian and Chinese scoring was largely unaffected by frequency of consumption, with significant differences only observed for juiciness. Daily consumers of lamb within Australia scored LL juiciness 16.8 to 22.5 units higher than those in lower frequency categories, similarly Chinese consumers who more frequently eat lamb scored juiciness 11.4 to 11.7 units higher than those that never eat lamb (*P* < 0.05; Table 2 and Table 5). As for the SM, the more frequent consumption habits (daily consumers) of Australian consumers resulted in higher scoring for tenderness (15.1–17.7 units), flavour (13.9–14.3 units) and overall liking (20.4–26.5 units) than those who had lower consumption habits. Similarly, Chinese consumers with greater consumption frequency habits scored tenderness (9.6–19.7 units), juiciness (3.1–17.2 units), flavour (11.7–20.4 units) and overall liking (4.3–17.2 units) higher than those in lower consumption categories. Within the USA, consumers that eat lamb monthly consistently scored LL tenderness, juiciness, flavour and overall liking (4.9–8.4 units) higher than those in categories of more and less frequent consumption. In contrast to Australian and Chinese consumers, the highest American consumption group (once per week) scored LL flavour and overall liking (10.1–18 units) lower than those that eat lamb less frequently. SM scoring was unaffected by frequency of consumption across all eating quality traits.

## 4. Discussion

### 4.1. The Effect of Consumer Age Group

In agreement with our hypothesis, consumer age had an impact on all eating quality traits, with older consumers generally scoring samples more favourably than younger consumers, particularly American and Chinese. For Chinese consumers, the tenderness scores of those that were 40 and above were higher across both cuts compared to younger age groups. For juiciness, flavour and overall liking scores a similar pattern was observed, although in this case, it did show some variation across cuts. American consumer tenderness scoring was similar to the Chinese, with those aged above 30 scoring tenderness higher than younger American consumers across both cuts. This trend was also evident for juiciness, flavour and overall liking, yet similar to the Chinese, this also varied across both cuts. In contrast, Australians scored tenderness lower amongst older consumers in both cuts, with the same trend present for juiciness, flavour and overall liking scores in the SM. The reverse was observed for the LL, whereby older consumers tended to score higher than some younger age groups (Table 3).

The results for American and Chinese consumers generally align with previous research in sheepmeat [14], where Australian consumers ≥31 years old scored tenderness 11.5 units, and juiciness 9.5 units higher than younger counterparts. In addition, Thompson et al. [9] also found Australians 40 to 50 years old scored juiciness 3.5 units higher than 20 to 25 year olds. The magnitude of difference between age categories within this study was comparable to the findings of Hastie et al. [14] and Thompson et al. [9], however, in this study, the impact of age also extended to flavour and overall liking. The consistency of the age group effect across tenderness, juiciness, flavour and overall liking was expected as these traits are all highly correlated, an association shown in this dataset [5] and numerous previous studies [21,22,23]. Nonetheless, demographic factors do not routinely impact on all eating quality traits [9,11,14]. Interestingly, Australian consumer scores did not align neatly with previous Australian findings of Hastie et al. [14] and Thompson et al. [9]. Discrepancies with previous results could be due to the larger sample size used in the current study compared to Hastie et al. [14] (*n* = 720 versus *n* = 75), or due to generational changes in consumer attitudes over time, given our testing was conducted at least ten years after Thompson et al. [9]. Alternatively, in contrast to the current findings, there are also studies where there was negligible or no effect of consumer age on eating quality scores in beef for Australian, French, Irish, Korean, Northern Irish and Polish consumers [10,11]. The differences found between age groups in the current study suggests tasting sessions should be balanced across age groups to ensure the eating quality preferences represented for the population of interest are not biased. Notably, differences found between Australian consumers in the current study and those of previous studies highlight the importance of testing large numbers of consumers, as well as continued testing over time to capture current population preferences.

### 4.2. The Effect of Number of Adults and Children in a Household

The number of adults in a household had a significant impact on average scores of all sensory traits, while the number of children in a household specifically affected tenderness (*P* < 0.05; Table 2). Participants from American and Australian households largely comprised of one to three adults, while Chinese consumers mainly came from households of three or above, which is consistent with traditional Chinese living arrangements of housing shared with extended family. In each country, a large proportion of consumers had 2 or less children, particularly in America and China, which is perhaps unsurprising given a large proportion of participants were aged 20–25 years old in these countries. When averaged across countries and muscle types, households with three adults consistently scored more favourably than households of more and less adults (up to 3.3 units; *P* < 0.05). This did however differ by country and muscle for tenderness and juiciness, for example higher tenderness scores corresponded with more adults in a household for Australian and Chinese consumers but not American (Table 2). Results partially align with previous research, whereby more adults in a household yielded higher sensory scores, however in the current study the effect on sensory traits varied somewhat and the magnitude of difference was much higher compared to Bonny et al. [10] (tenderness and overall liking, range 0.5–1.0 units) and Thompson et al. [9] (juiciness, range 1.7 to 3.7 units). The effect of the number of children in a household, was restricted to tenderness scores of the SM samples for American consumers, where consumers of households of 5 children scored higher than those of 0 to 3 children (up to 16.1 units; *P* < 0.05). This particular sub-class consisted of only 9 consumers, and as such should be interpreted with caution. Country differences were also observed in scoring for tenderness and juiciness within the adult and children sub-classes. Overall, American and Australian scores were higher than Chinese consumers (*P* < 0.05), these particular subgroup differences may help explain some drivers of the country effect observed for tenderness in the production factor analysis of the same dataset [5].

### 4.3. The Effect of Consumer Gender

In support of our hypothesis, gender had an impact on eating quality scores but was only evident in American consumers for flavour and overall liking traits. American males scored flavour and overall liking around 3.5 units higher than females. This aligns with previous research of Bonny et al. [10] and Kubberød et al. [13] with higher scores observed for males within some countries tested, however the magnitude of effect was almost double in this study compared to Bonny et al. [10]. Some speculation surrounding gender scoring differences, has attributed higher scores to a greater appreciation of meat in the diet, for males in particular [10,13]. Our findings may support this, as there were ten percent more American males than females (Table 1), and a higher proportion of males were more frequent lamb consumers and higher appreciators of lamb compared to females (data not shown). Notably, previous research by Huffman et al. [12] found no impact of gender on sensory scoring for American consumers. Similarly, within our study, there was no difference in scoring between the genders for Australian and Chinese consumers, which aligns with Hwang et al. [11] testing Australian and Korean sensory responses to grilled and Korean barbequed beef. Chinese females tended towards lower consumption and appreciation of lamb in the diet compared to males, and with around twenty percent more females recruited within the China cohort, a possible negative gender effect was expected but was not apparent. These results are in contrast to findings of Thompson et al. [9] and Hastie et al. [14] demonstrating an improvement in scoring for Australian females compared to males. Lack of a consistent gender effect across the countries suggests the differences found may be a reflection of other societal or inherent red meat consumption preferences, rather than gender alone. However, regardless of the driver, eating quality preferences particular to gender in America were demonstrated and as such require consideration when evaluating palatability of sheepmeat.

### 4.4. The Effect of Consumer Income

Validating our hypothesis, income bracket had an impact on average sensory scores for tenderness, flavour and overall liking, and differed by country and muscle for flavour and overall liking (*P* < 0.05; Table 2). The most prominent trend was within Australian consumers, with lower income earners scoring more favourably than higher income earners except for those earning above $150,000 per annum (up to 9.3 units higher; *P* ≤ 0.05; Table 4). This result is consistent with Huffman et al. [12] who demonstrated higher income earners to score sensory traits lower when conducting in home evaluations of beef, with the suggestion that product expectations may be greater in higher income earners. It stands to reason that higher income earners may have easier access to protein sources and premium products, therefore be more frequent consumers and thus more discerning. However, for Australian consumers, distribution of participants across consumption frequency and lamb appreciation categories was very consistent across all income brackets (data not shown), demonstrating Australian income groups were homogenous in regards to consumption preferences.

In contrast to Australian results, there was no consistent impact of income on sensory scoring for Chinese consumers. A tenderness difference was detected for the group who did not want to divulge their income compared to one income bracket, as thus no conclusion can be drawn. Flavour scores increased with rising income up to ¥96,000 for the LL and were higher only for income bracket ¥24,000 to ¥36,000 in the SM, similarly, overall liking increases were restricted to the ¥24,000 to ¥36,000 bracket for the LL and no difference in the SM. Mao et al. [8] reported that in urban areas, income is a strong driver of consumption frequency of sheepmeat with positive attitude changes to sheepmeat accompanying increased income categories. However, within this study, there were no marked differences in frequency of consumption distribution across the income brackets measured (data not shown). Following the same trend as Chinese scoring, there was no consistent pattern of scoring for American consumers across income brackets. Tenderness was scored most favourably by those earning US$75,000 to $100,000 per annum, higher flavour scores were reported for those earning under US$50,000 and US$75,000 to $100,000, while overall liking increases were restricted to those earning under US$50,000 (up to 4.9 units higher; *P* ≤ 0.05; Table 4). This is in partial agreement with Huffman et al. [12] whereby lower income earners scored tenderness, juiciness and flavour more favourably than higher income earners. Income had a significant influence on eating quality scores for all three countries. As such, sensory panels should strive for a balanced representation of different incomes, achieved through sampling of large numbers of consumers and inclusion of different geographic locations.

### 4.5. The Effect of Meat Consumption Habits

Contrary to our hypothesis, appreciation of lamb in the diet and preferred degree of cooking doneness did not have an impact on eating quality scores within this study. In contrast to previous studies [9,10,11,21], the only meat consumption habit to influence sensory scores was frequency of consumption, which was significant for all sensory traits and varied across muscles (Table 1 and Table 2). Examination of the number of lamb appreciators spread across consumption frequency categories, showed those that selected highest consumption rates were largely the highest appreciators and vice versa (data not shown). As such, it appears consumption habits and attitudes are largely intertwined. Higher frequency of consumption had positive effect on juiciness scores in the LL for Australian and Chinese consumers (*P* ≤ 0.05; Table 5), and tenderness, juiciness, flavour and overall liking in the SM (*P* ≤ 0.05; Table 5). These results should be interpreted with caution as significant differences were detected mainly between the highest and lowest consumption categories within Australian and Chinese consumer groups (these being, daily consumers within Australia (n = 5) and those that never eat lamb in China (*n* = 15); Table 1). Therefore, these results are unlikely to be representative of the wider population.

For American consumers, frequency of consumption had no impact on scores of the SM, however monthly consumers of lamb (*n* = 64) scored LL tenderness, juiciness, flavour and overall liking up to 8.4 units greater than more and less frequent consumers (*P* ≤ 0.05; Table 5). Given previous studies have demonstrated a greater appreciation of red meat to impact on sensory scores [9,10,21], a suggestion for more favourable scores in the American once a month category is that they have a greater proportion of higher appreciators than every other category. Monthly consumption of lamb would likely be considered a higher frequency category in the USA, given annual per capita consumption rates are quite low [6]. Therefore, while close to 70% of monthly consumers identified lamb as important in their diet (data not shown), the weekly consumption category who scored LL flavour and overall liking lower, actually had a higher proportion of appreciators, negating this theory. This negative response observed in the most frequent consumers of lamb in the American cohort (weekly; Table 1) could be attributed to a preference for locally sourced lamb, as more favourable eating quality scores have been observed for domestic products compared to imported Australian lamb [24]. However, similar to Australia and China, consumer numbers were very low (*n* = 10) for this group and as such should be interpreted with a degree of caution.

## 5. Conclusions

Confirming our hypothesis, demographic factors had a variable impact on eating quality scores of Australian, Chinese and American consumers testing Australian lamb and yearling samples grilled according to MSA protocols. Demographic attributes of consumer age, gender and number of adults in a household, and income bracket had a significant but different effect within the three countries, occasionally varying by muscle and sensory trait. There was minimal effect of the number of children in a household and no effect of consumer occupation on sensory scores. Contrary to our hypothesis, the importance of sheepmeat and preferred degree of cooking doneness did not impact on eating quality scores for these consumer groups. Frequency of consumption had a significant effect in all three countries, contrasting with previous studies examining the effects of meat consumption habits on sensory scores. However, under-represented consumption categories at either end of the spectrum were largely driving results in this study. Overall, the magnitude of effect for significant attributes in this study were generally greater than those previously reported. This suggests that sensory panels within Australia, China and the USA should be balanced where possible, for demographic factors of age, gender, number of adults in a household and income, with the aim of reducing any bias on sheepmeat sensory scores. Challenges of bias can be overcome through recruitment of sufficiently large populations, and inclusion of geographically diverse locations to help ensure the most accurate representation of sensory preferences is captured for the population of interest.

## Figures and Tables

**Table 1:** Percentage distribution of consumers who scored sheepmeat samples (and number of consumers) within each demographic and meat consumption category for each country.

Consumer Age	<20	20–25	26–30	31–39	40–60	>60		
Australia	7.64 (55)	8.33 (60)	6.94 (50)	19.86 (143)	49.72 (358)	7.50 (54)		
China	6.39 (46)	48.75 (351)	19.31 (139)	4.31 (31)	12.50 (90)	8.75 (63)		
USA	10.39 (70)	41.25 (278)	9.20 (62)	11.57 (78)	23.00 (155)	4.60 (31)		
**Gender**	**Male**	**Female**						
Australia	54.17 (390)	45.83 (330)						
China	37.22 (268)	62.78 (452)						
USA	55.04 (371)	44.96 (303)						
**Adults**	**1**	**2**	**3**	**4**	**≥5**			
Australia	7.36 (53)	51.11 (368)	20.14 (145)	14.72 (106)	6.67 (48)			
China	2.50 (18)	15.00 (108)	42.22 (304)	25.14 (181)	15.14 (109)			
USA	16.34 (110)	45.77 (308)	21.4 (144)	10.40 (70)	6.09 (41)			
**Children**	**0**	**1**	**2**	**3**	**4**	**≥ 5**		
Australia	34.03 (245)	20.28 (146)	26.67 (192)	12.64 (91)	3.75 (27)	2.64 (19)		
China	69.31 (499)	23.89 (172)	5.00 (36)	1.39 (10)	0.28 (2)	0.14 (1)		
USA	62.17 (419)	15.43 (104)	11.42 (77)	7.27 (49)	2.37 (16)	1.34 (9)		
**Income level ^1^**	***a***	***b***	***c***	***d***	***e***	***f***	***g***	***h***
Australia	6.39 (46)	10.42 (75)	14.58 (105)	17.36 (125)	13.61 (98)	10.97 (79)	15.28 (110)	11.39 (82)
China	48.33 (348)	15.97 (115)	12.08 (87)	8.19 (59)	2.64 (19)	4.44 (32)	8.33 (60)	
USA	19.88 (134)	19.88 (134)	19.14 (129)	17.95 (121)	23.15 (156)			
**Occupation**	**Manager**	**Professional**	**Trade Worker**	**Community Service**	**Administration**	**Sales and Service**		
Australia	17.36 (125)	22.22 (160)	13.06 (94)	4.58 (33)	8.75 (63)	3.06 (22)		
China	3.75 (27)	20.97 (151)	9.86 (71)	1.81 (13)	5.42 (39)	3.06 (22)		
USA		6.23 (42)	6.82 (46)		28.64 (193)	0.74 (5)		
**Occupation**	**Machinery Operators**	**Labourer**	**Home Duties**	**Student**	**Other**			
Australia	5.56 (40)	3.47 (25)	5.00 (36)	5.28 (38)	11.67 (84)			
China	1.81 (13)	10.69 (77)	0.83 (6)	28.75 (207)	13.06 (94)			
USA		10.83 (73)	32.94 (222)	8.61 (58)	5.19 (35)			
**Frequency of Lamb Consumption ^2^**	***a***	***b***	***c***	***d***	***e***	***f***	***g***	
Australia	0.69 (5)	1.94 (14)	14.31 (103)	36.11 (260)	23.61 (170)	22.64 (163)	0.69 (5)	
China	0.83 (6)	1.39 (10)	12.36 (89)	21.81 (157)	24.44 (176)	37.08 (267)	2.08 (15)	
USA	0 (0)	1.48 (10)	4.01 (27)	9.50 (64)	8.61 (58)	42.88 (289)	33.53 (226)	
**Importance of Lamb in the Diet**	**Appreciator of lamb**	**Lamb is important**	**Indifferent to lamb**	**Rarely/never eat lamb**				
Australia	29.44 (212)	44.17 (318)	24.31 (175)	2.08 (15)				
China	5.69 (41)	29.03 (209)	62.08 (447)	3.19 (23)				
USA	7.27 (49)	14.54 (98)	35.16 (237)	43.03 (290)				
**Preferred Degree of Doneness**	**Blue**	**Rare**	**Medium/Rare**	**Medium**	**Medium/Well Done**	**Well Done**		
Australia	0.28 (2)	3.33 (24)	32.78 (236)	28.19 (203)	25.28 (182)	10.14 (73)		
China	0 (0)	0 (0)	0.28 (2)	1.94 (14)	23.75 (171)	74.03 (533)		
USA	0 (0)	4.01 (27)	30.12 (203)	27.00 (182)	29.23 (197)	9.64 (65)		

^1^ Income categories different for each country. In all countries, income level per annum. Australia (AUD) *(a)* <$25,000, *(b)* $25,000–$50,000, *(c)* $50,001–$75,000, *(d)* $75,001–$100,000, *(e)* $100,001–$125,000, *(f)* $125,001–$150,000, *(g)* > $150,000 *(h)* Prefer not to say; China (yuan): *(a)* ≤¥24,000, *(b)* ¥24,000–¥36,000, *(c)* ¥36,001–¥60,000, *(d)* ¥60,001–¥96,000, *(e)* ¥96,001–¥120,000, *(f)* > ¥120,000, *(g)* Prefer not to say; and the USA (USD): *(a)* < $20,000, *(b)* $20,000–$50,000, *(c)* $50,001–$75,000, *(d)* $75,001–$100,000, *(e)* >$100,000’; ^2^ Consumption frequency categories different for the USA. For Australia and China (a) daily, (b) 4–5 times per week, (c) 2–3 times per week, (d) weekly, (e) fortnightly, (f) monthly, (g) never eat lamb; for the USA (a) daily, (b) weekly, (c) fortnightly, (d) monthly, (e) every other month, (f) 2–3 times per year, (g) never eat lamb.

**Table 2:** *F*-values, numerator and denominator degrees of freedom (NDF and DDF) for base linear mixed effects models for predicted eating quality scores of tenderness, and juiciness, liking of flavour and overall liking.

	Tenderness		Juiciness		Flavour		Overall Liking	
Effects	NDF, DDF	*F*-Value	NDF, DDF	*F*-Value	NDF, DDF	*F*-Value	NDF, DDF	*F*-Value
Country	2, 2107	2.50	2, 2225	2.48	2, 2083	3.42 *	2, 2235	1.41
Muscle	1, 9944	69.12 **	1, 10,000	105.97 **	1, 10,000	104.85 **	1, 9978	146.96 **
Country*muscle	2, 9963	1.29	2, 9978	3.39 *	2, 9997	0.84	2, 9984	0.43
Age	5, 2029	5.45 **	5, 2060	2.01	5, 2055	2.80 *	5, 2054	3.49 *
Gender	–	–	1, 2063	0.14	1, 2061	2.49	1, 2058	2.45
Adults	4, 2021	3.25 *	4, 2050	3.90 *	4, 2052	3.52 *	4, 2048	3.46 *
Children	5, 2001	0.38	–	–	–	–	–	–
Income level (country)	17, 2021	2.68 **	–	-	17, 2052	1.88 *	17, 2047	2.13 *
Consumption frequency (country)	17, 2024	0.96	17, 2053	1.21	17, 2054	1.06	17, 2053	1.25
Country*age	10, 2032	2.58 *	10, 2063	3.01 **	10, 2058	2.55 *	10, 2058	2.36 *
Country*gender	–	–	–	–	2, 2062	4.71 *	2, 2058	3.88 *
Country*adults	8, 2023	1.11	8, 2051	2.76 *	–	–	–	–
Country*children	10, 2024	0.90	–	–	–	–	–	-
Muscle*age	5, 9961	2.67 *	5, 9990	3.36 *	5, 10,000	1.89 *	5, 9938	1.70
Muscle*gender	–	–	1, 10,000	6.77 *	–	–	–	–
Muscle*adults	4, 9957	0.88	–	–	–	–	–	–
Muscle*children	5, 9942	1.17	–	–	–	–	–	–
Muscle*income level (country)	–	–	–	–	17, 10,000	1.69 *	17, 9929	1.94 *
Muscle*consumption frequency (country)	17, 9952	2.71 **	17, 9989	1.71 *	17, 10,000	2.11 *	17, 9935	2.37 *
Country*muscle*adults	8, 9961	4.15 **	–	–	–	–	–	–
Country*muscle*children	10, 9957	2.96 *	–	–	–	–	–	–
Country*muscle*age	–	–	10, 9987	2.02 *	10, 10,000	2.67 *	10, 9934	3.30 **

*: *P* < 0.05: **: *P* < 0.001; –: effect not in final base model after stepwise regression.

**Table 3:** Least square means ± standard error (on a scale of 0–100) of tenderness, juiciness, flavour and overall liking scores of m. *longissimus lumborum* (LL) and m. *semimembranosus* (SM) samples by a consumer’s country and age group. Values within brackets signify difference from mean of country and muscle type (*italics = greater than 3 units from the mean*).

Sensory Trait	Tenderness		Juiciness		Flavour		Overall Liking	
Muscle Type	LL	SM	LL	SM	LL	SM	LL	SM
**Age (years)**	*Australian consumers*							
<20	69.0 ± 2.8 ^a^ (1.1)	64.4 ± 2.8 ^a^ *(7.8*)	70.0 ± 2.6 ^abc^ (0.5)	65.7 ± 2.6 ^b^ (*5.6*)	71.4 ± 2.6 ^ab^ (2.3)	66.5 ± 2.6 ^a^ (*5.7*)	72.4 ± 2.6 ^b^ (2.3)	68.2 ± 2.6 ^d^ (*7.0*)
20–25	66.9 ± 2.9 ^ac^ (−1.0)	59.2 ± 2.9 ^ac^ (2.6)	69.6 ± 2.6 ^abc^ (0)	64.3 ± 2.6 ^b^ (*4.2*)	69.3 ± 2.5 ^ab^ (0.2)	65.9 ± 2.5 ^ab^ (*5.1*)	69.9 ± 2.6 ^ab^ (−0.2)	65.5 ± 2.6 ^cd^ (*4.4*)
26–30	65.4 ± 3.0 ^b^ (−2.5)	49.9 ± 3.0 ^b^ (−*6.7*)	67.9 ± 2.7 ^ab^ (−1.6)	55.1 ± 2.7 ^a^ (−*5.0*)	65.6 ± 2.6 ^a^ (−*3.6*)	54.0 ± 2.7 ^d^ (−*6.8*)	66.2 ± 2.7 ^a^ (−*3.9*)	53.6 ± 2.7 ^a^ (−*7.5*)
31–39	69.3 ± 2.3 ^bc^ (1.4)	53.8 ± 2.3 ^bc^ (−2.9)	70.4 ± 2.1 ^bc^ (0.9)	58.5 ± 2.1 ^a^ (−1.7)	69.9 ± 2.0 ^ab^ (0.7)	59.2 ± 2.0 ^c^ (−1.6)	70.9 ± 2.0 ^ab^ (0.8)	59.5 ± 2.1 ^b^ (−1.7)
40–60	68.0 ± 2.1 ^bc^ (0.2)	55.5 ± 2.1 ^bc^ (−1.2)	66.3 ± 1.8 ^a^ (−*3.2*)	57.3 ± 1.8 ^a^ (−2.8)	66.9 ± 1.7 ^a^ (−2.2)	58.9 ± 1.8 ^c^ (−2.0)	68.2 ± 1.8 ^ab^ (−1.9)	59.7 ± 1.8 ^b^ (−1.4)
>60	68.6 ± 3.1 ^ac^ (0.7)	57.0 ± 3.2 ^ac^ (0.4)	73.0 ± 2.7 ^c^ (*3.4*)	59.9 ± 2.7 ^ab^ (−0.2)	71.7 ± 2.7 ^b^ (2.6)	60.4 ± 2.7 ^bc^ (−0.5)	72.7 ± 2.7 ^b^ (2.7)	60.4 ± 2.7 ^bc^ (−0.7)
	*Chinese consumers*							
<20	60.5 ± 4.6 ^a^ (−*3.5*)	47.6 ± 4.6 ^a^ (−*4.2*)	61.0 ± 2.7 ^ab^ (−1.8)	54.7 ± 2.7 ^b^ (−2.5)	63.4 ± 2.9 (−1.6)	49.7 ± 2.9 ^a^ (−*6.3*)	66.4 ± 2.9 (−0.9)	51.2 ± 2.9 ^a^ (−*5.5*)
20–25	63.2 ± 4.1 ^a^ (−0.8)	51.0 ± 4.1 ^a^ (−0.7)	61.8 ± 1.7 ^a^ (−1.0)	56.6 ± 1.7 ^b^ (−0.6)	64.2 ± 1.9 (−0.8)	53.5 ± 1.9 ^ac^ (−2.6)	67.2 ± 2.0 (−0.1)	54.4 ± 2.0 ^a^ (−2.2)
26–30	63.7 ± 4.0 ^a^ (−0.2)	51.0 ± 4.0 ^a^ (−0.8)	64.2 ± 2.0 ^ab^ (1.4)	57.9 ± 2.0 ^ab^ (0.7)	65.4 ± 1.9 (0.4)	55.5 ± 1.9 ^bc^ (−0.5)	67.1 ± 2.0 (−0.2)	56.6 ± 2.0 ^ab^ (0)
31–39	61.9 ± 4.7 ^a^ (−2.1)	47.4 ± 4.7 ^a^ (−*4.4*)	59.9 ± 3.2 ^ab^ (−2.9)	53.2 ± 3.2 ^b^ (−4)	65.0 ± 3.1 (−0.1)	55.9 ± 3.1 ^acd^ (−0.1)	66.7 ± 3.1 (−0.6)	56.1 ± 3.1 ^ab^ (−0.6)
40–60	67.1 ± 4.0 ^b^ (*3.2*)	55.6 ± 4.0 ^b^ (*3.9*)	66.0 ± 2.1 ^b^ (*3.2*)	59.6 ± 2.1 ^ab^ (2.4)	65.5 ± 2.1 (0.4)	60.3 ± 2.1 ^d^ (*4.2*)	67.9 ± 2.2 (0.6)	60.4 ± 2.2 ^b^ (*3.7*)
>60	67.4 ± 4.3 ^b^ (*3.4*)	58.0 ± 4.3 ^b^ (*6.2*)	63.8 ± 2.4 ^ab^ (1)	61.0 ± 2.5 ^ac^ (*3.9*)	66.7 ± 2.5 (1.6)	61.4 ± 2.5 ^d^ (*5.4*)	68.6 ± 2.5 (1.3)	61.4 ± 2.5 ^b^ (*4.7*)
	*American consumers*							
<20	72.0 ± 2.5 ^a^ (−1.2)	56.4 ± 2.6 ^a^ (−1.1)	67.9 ± 2.2 ^abc^ (−0.4)	57.8 ± 2.2 ^ab^ (−0.3)	69.5 ± 2.1 ^ab^ (1.5)	64.4 ± 2.1 ^ac^ (*3.2*)	70.0 ± 2.2 ^ab^ (1.4)	62.0 ± 2.2n^b^ (2.9)
20–25	69.6 ± 2.0 ^a^ (−*3.7*)	55.1 ± 2.0 ^a^ (−2.4)	65.3 ± 1.4 ^a^ (−2.9)	57.6 ± 1.4 ^a^ (−0.4)	65.6 ± 1.4 ^a^ (−2.3)	59.7 ± 1.4 ^bd^ (−1.4)	65.8 ± 1.4 ^a^ (−2.7)	57.4 ± 1.4 ^ac^ (−1.7)
26–30	72.2 ± 2.8 ^a^ (−1.1)	52.2 ± 2.8 ^a^ (−*5.4*)	65.6 ± 2.3 ^ab^ (−2.6)	52.8 ± 2.3 ^b^ (−*5.2*)	65.5 ± 2.2 ^ab^ (−2.5)	56.4 ± 2.2 ^b^ (−*4.8*)	65.9 ± 2.3 ^a^ (−2.7)	53.1 ± 2.3 ^a^ (−*6.0*)
31–39	72.5 ± 2.5 ^a^ (−0.7)	56.8 ± 2.5 ^a^ (−0.7)	68.4 ± 2.2 ^abc^ (0.2)	59.1 ± 2.2 ^a^ (1.1)	67.2 ± 2 ^ab^ (−0.7)	60.5 ± 2.0 ^bcd^ (−0.6)	68.1 ± 2.1 ^ab^ (−0.5)	59.3 ± 2.1 ^bc^ (0.2)
40–60	76.4 ± 2.3 ^b^ (*3.2*)	60.5 ± 2.3 ^b^ (3.0)	70.9 ± 1.8 ^bc^ (2.6)	59.3 ± 1.8 ^a^ (1.2)	70.0 ± 1.7 ^b^ (2.0)	61.8 ± 1.7 ^acd^ (0.7)	70.9 ± 1.7 ^b^ (2.4)	59.7 ± 1.7 ^bc^ (0.6)
>60	76.7 ± 3.6^b^ (*3.5*)	64.3 ± 3.7 ^b^ (*6.7*)	71.3 ± 3.1 ^c^ (*3.1*)	61.5 ± 3.2 ^a^ (*3.5*)	70.0 ± 3.0 ^ab^ (2.0)	64.1 ± 3.0 ^acd^ (3.0)	70.6 ± 3.1 ^ab^ (2.0)	63.1 ± 3.1 ^bc^ (*4.0*)

^a, b, c, d^ Values within a column, country and muscle type with different superscript letters, differ significantly at *P* < 0.05.

**Table 4:** Least square means ± standard error (on a scale of 0–100) of tenderness, juiciness, flavour and overall liking scores of sheepmeat samples by a consumer’s income bracket. Values within brackets signify difference from mean of country (*italics = greater than 3 units from the mean*).

Sensory Trait	Tenderness	Juiciness	Flavour	Overall Liking
**Income** AUD	*Australian consumers*			
<$25,000	63.9 ± 2.6 ^abc^ (1.6)	67.3 ± 2.5 (2.8)	66.0 ± 2.4 ^ab^ (1.0)	67.4 ± 2.4 ^bc^ (1.8)
$25,000–$50,000	67.9 ± 2.3 ^a^ (*5.7*)	66.6 ± 2.2 (2.1)	68.4 ± 2.1 ^b^ (*3.4)*	70.3 ± 2.1 ^c^ (*4.7)*
$50,001–$75,000	64.1 ± 2.1 ^ab^ (1.8)	65.8 ± 2.0 (1.3)	65.8 ± 1.9 ^ab^ (0.8)	66.6 ± 2.0 ^bc^ (1.0)
$75,001–$100,000	63.1 ± 2.1 ^bc^ (0.9)	63.5 ± 2.0 (−1.0)	63.8 ± 1.9 ^a^ (−1.2)	64.4 ± 1.9 ^ab^ (−1.2)
$100,001–$125,000	58.6 ± 2.3 ^d^ (*−3.6*)	61.9 ± 2.1 (−2.7)	62.1 ± 2.0 ^a^ (−2.9)	62.2 ± 2.1 ^a^ (*−3.4*)
$125,001–$150,000	60.1 ± 2.4 ^bcd^ (−2.1)	63.1 ± 2.3 (−1.5)	64.4 ± 2.2 ^ab^ (−0.6)	64.1 ± 2.2 ^ab^ (−1.5)
>$150,000	62.4 ± 2.2 ^bc^ (0.1)	64.4 ± 2.1 (−0.1)	65.4 ± 2.0 ^ab^ (0.4)	65.7 ± 2.0 ^ab^ (0.1)
Prefer not to say	57.9 ± 2.3 ^d^ (*−4.4*)	63.6 ± 2.1 (−0.9)	64.0 ± 2.1 ^a^ (−1.0)	64.1 ± 2.1 ^ab^ (−1.5)
Yuan	*Chinese consumers*			
≤¥24,000	57.3 ± 3.3 ^ab^ (−0.6)	59.5 ± 1.8 (−0.2)	60.4 ± 1.7 ^ab^ (−0.2)	61.3 ± 1.7 ^a^ (−0.7)
¥24,000–¥36,000	59.3 ± 3.5 ^ab^ (1.4)	62.3 ± 1.9 (2.6)	62.8 ± 1.8 ^a^ (2.3)	64.7 ± 1.8 ^b^ (2.7)
¥36,001–¥60,000	57.5 ± 3.6 ^ab^ (−0.4)	59.4 ± 2 (−0.3)	58.0 ± 1.9 ^b^ (−2.5)	60.0 ± 1.9 ^a^ (−2.0)
¥60,001–¥96,000	61.4 ± 3.7 ^a^ (*3.5*)	63.5 ± 2.3 (*3.8*)	63.4 ± 2.2 ^a^ (2.8)	64.1 ± 2.2 ^ab^ (2.1)
¥96,001–¥120,000	55.5 ± 4.6 ^ab^ (−2.4)	57.7 ± 3.5 (−2.0)	57.8 ± 3.4 ^ab^ (−2.7)	61.0 ± 3.5 ^ab^ (−1.0)
>¥120,000	58.7 ± 4.1 ^ab^ (0.8)	56.5 ± 2.9 (*−3.2*)	61.5 ± 2.8 ^ab^ (0.9)	62.8 ± 2.8 ^ab^ (0.8)
Prefer not to say	55.4 ± 3.6 ^b^ (−2.5)	59.0 ± 2.3 (−0.7)	59.9 ± 2.2 ^ab^ (−0.6)	60.2 ± 2.3 ^ab^ (−1.8)
USD	*American consumers*			
<$20,000	67.3 ± 2.2 ^ab^ (1.9)	65.2 ± 1.8 (2.0)	66.4 ± 1.7 ^a^ (1.9)	66.3 ± 1.7 ^a^ (2.5)
$20,000–$50,000	64.6 ± 2.0 ^ab^ (−0.8)	63.2 ± 1.7 (0.1)	66.0 ± 1.5 ^a^ (1.5)	65.2 ± 1.6 ^a^ (1.4)
$50,001–$75,000	64.0 ± 2.0 ^a^ (−1.5)	62.0 ± 1.7 (−1.2)	62.0 ± 1.6 ^b^ (−2.5)	61.6 ± 1.6 ^bc^ (−2.2)
$75,001–$100,000	67.4 ± 2.0 ^b^ (2.0)	64.0 ± 1.7 (0.8)	66.1 ± 1.6 ^a^ (1.5)	64.6 ± 1.6^ac^ (0.7)
>$100,000	63.8 ± 1.9 ^a^ (−1.6)	61.4 ± 1.6 (−1.8)	62.2 ± 1.6 ^b^ (−2.3)	61.4 ± 1.6 ^bc^ (−2.4)

^a, b, c, d^ Values within a column and country with different superscript letters, differ significantly at *P* < 0.05.

**Table 5:** Least square means ± standard error (on a scale of 0–100) of tenderness, juiciness, flavour and overall liking scores of LL and SM samples by a consumer’s consumption frequency of sheepmeat. Values within brackets signify difference from mean of country and muscle type (*italics = greater than 3 units from the mea*n).

Sensory Trait	Tenderness		Juiciness		Flavour		Overall Liking	
Muscle Type	LL	SM	LL	SM	LL	SM	LL	SM
**Consumption Frequency**	*Australian consumers*							
Daily	72.5 ± 7.4 (*4.5)*	69.6 ± 7.3 ^b^ (*13.1*)	85.1 ± 7.2 ^b^ (*15.5*)	70.0 ± 7.1 (*9.9)*	77.6 ± 7.1 (*8.4*)	72.8 ± 7.0 ^b^ (*12.0*)	79.7 ± 7.2 (*9.6*)	81.0 ± 7.1 ^b^ (*19.9*)
4–5/week	63.7 ± 4.5 (*−4.3)*	53.4 ± 4.5 ^a^ (*−3.1*)	62.6 ± 4.3 ^a^ (*−7.0*)	55.2 ± 4.3 (*−5.0*)	63.3 ± 4.2 (*−5.9*)	60.3 ± 4.2 ^ab^ (−0.5)	65.7 ± 4.3 (*−4.4*)	60.6 ± 4.3 ^a^ (−0.5)
2–3/week	68.7 ± 1.9 (0.8)	55.4 ± 1.9 ^a^ (−1.1)	68.3 ± 1.7 ^a^ (−1.3)	59.6 ± 1.7 (−0.5)	69.7 ± 1.7 (0.6)	60.4 ± 1.7 ^ab^ (−0.4)	70.8 ± 1.7 (0.7)	59.4 ± 1.7 ^a^ (−1.7)
1/week	67.7 ± 1.6 (−0.2)	51.9 ± 1.6 ^a^ (*−4.6*)	67.3 ± 1.2 ^a^ (−2.3)	57.1 ± 1.2 (−3)	69.6 ± 1.2 (0.5)	58.5 ± 1.1 ^a^ (−2.3)	70.3 ± 1.2 (0.2)	56.8 ± 1.2 ^a^ (*−4.4*)
Fortnightly	68.6 ± 1.7 (0.6)	51.9 ± 1.7 ^a^ (*−4.6*)	67.6 ± 1.4 ^a (^−1.9)	57 ± 1.4 (*−3.1*)	68.4 ± 1.3 (−0.8)	58.9 ± 1.3 ^a^ (−2.0)	70 ± 1.4 (0)	56.9 ± 1.4 ^a^ (*−4.2*)
Monthly	66.9 ± 1.7 (−1.1)	54.5 ± 1.7 ^a^ (−2.0)	67.1 ± 1.4 ^a^ (−2.4)	59.6 ± 1.4 (−0.5)	69.2 ± 1.3 (0.1)	59.5 ± 1.3 ^ab^ (−1.3)	69.6 ± 1.4 (−0.4)	58.7 ± 1.4 ^a^ (−2.4)
Never eat	67.8 ± 7.3 (*9.5*)	58.7 ± 7.6 ^ab^ (2.2)	68.9 ± 7.1 ^ab^ (7.6)	62.4 ± 7.3 (2.2)	66.2 ± 6.9 (*3.3*)	55.4 ± 7.2 ^ab^ (*−5.4*)	64.3 ± 7.0 (1.1)	54.6 ± 7.2 ^a^ (*−6.6*)
	*Chinese consumers*							
Daily	58.2 ± 7.5 (*−5.5*)	54.2 ± 7.6 ^abc^ (2.2)	55.1 ± 6.5 ^ab^ (*−7.7*)	56.4 ± 6.6 ^ab^ (−0.7)	65.1 ± 6.4 (0.1)	60.0 ± 6.5 ^a^ (*4.0*)	69.1 ± 6.4 ^ab^ (1.8)	62.3 ± 6.5 ^ac^ (*5.6)*
4–5/week	59.4 ± 6.3 (*−4.3*)	62.2 ± 6.3 ^c^ (*10.2*)	64.1 ± 5.1 ^ab^ (1.3)	67.0 ± 5.1 ^a^ (*9.8*)	62.6 ± 5.0 (−2.4)	63.6 ± 5.0 ^a^ (7.6)	67.2 ± 5.1 ^ab^ (−0.1)	64.0 ± 5.1 ^a^ (*7.3*)
2–3/week	67.6 ± 4.0 (*3.9*)	52.9 ± 3.9 ^bc^ (0.9)	66.5 ± 1.9 ^a^ (*3.7*)	57.6 ± 1.9 ^ab^ (0.4)	66.1 ± 1.8 (1.0)	57.9 ± 1.8 ^a^ (1.8)	68.0 ± 1.9 ^ab^ (0.6)	58.1 ± 1.9 ^a^ (1.4)
1/week	68.0 ± 3.7 (*4.3*)	49.2 ± 3.7 ^ab^ (−2.8)	66.6 ± 1.5 ^a^ (*3.8*)	55.5 ± 1.5 ^bc^ (−1.7)	67.2 ± 1.5 (2.1)	54.9 ± 1.5 ^a^ (−1.2)	68.7 ± 1.5 ^ab^ (1.3)	53.8 ± 1.5 ^bc^ (−2.9)
Fortnightly	67.0 ± 3.7 (*3.3*)	52.1 ± 3.7 ^bc^ (0.1)	66.3 ± 1.5 ^a^ (*3.4*)	58.5 ± 1.5 ^ac^ (1.4)	66.7 ± 1.4 (1.6)	57.6 ± 1.4 ^a^ (1.6)	69.1 ± 1.5 ^b^ (1.8)	56.6 ± 1.5 ^ac^ (−0.1)
Monthly	66.5 ± 3.7 (2.8)	51.0 ± 3.7 ^b^ (−1.0)	66.3 ± 1.3 ^a^ (*3.4*)	55.5 ± 1.3 ^b^ (−1.7)	66.6 ± 1.2 (1.6)	55.2 ± 1.2 ^a^ (−0.9)	68.4 ± 1.3 ^ab^ (1.1)	55.3 ± 1.3 ^ac^ (−1.4)
Never eat	59.3 ± 5.5 (*−4.4*)	42.5 ± 5.5^a^ (*−9.6*)	54.9 ± 4.3 ^b^ (*−7.9*)	49.7 ± 4.3 ^b^ (*−7.4*)	61 ± 4.2 (*−4.1*)	43.2 ± 4.2 ^b^ (*−12.8*)	60.8 ± 4.2 ^a^ (*−6.5*)	46.8 ± 4.2 ^b^ (*−9.9*)
	*American consumers*							
Daily	-	-	-	-	-	-	-	-
1/week	70.8 ± 5.3 ^ac^ (−2.4)	59.0 ± 5.3 (1.3)	62.8 ± 5.0 ^ab^ (*−5.5*)	57.7 ± 5.0 (−0.3)	59.4 ± 4.9 ^ac^ (*−8.6*)	60.8 ± 4.9 (−0.4)	56.3 ± 5.1 ^a^ (*−12.2*)	58.5 ± 5.0 (−0.6)
Fortnightly	72.9 ± 3.5 ^ac^ (−0.3)	57.5 ± 3.4 (−0.2)	69.1 ± 3.2 ^ab^ (0.9)	57.5 ± 3.2 (−0.5)	70.5 ± 3.1 ^bd^ (2.5)	60.3 ± 3.1 (−0.8)	72.3 ± 3.1 ^bc^ (*3.7*)	58.1 ± 3.1 (−1.0)
Monthly	78.1 ± 2.6 ^a^ (*4.9*)	58.8 ± 2.6 (1.1)	72.6 ± 2.1 ^a^ (*4.4)*	58.4 ± 2.2 (0.4)	72.8 ± 2.1 ^b^ (*4.9*)	61.4 ± 2.1 (0.3)	74.3 ± 2.1 ^b^ (*5.8*)	59.8 ± 2.1 (0.7)
Every other month	69.6 ± 2.7 ^bc^ (*−3.5*)	57.4 ± 2.7 (−0.3)	66.6 ± 2.3 ^b^ (−1.6)	57.1 ± 2.3 (−0.9)	68.7± 2.1 ^abc^ (0.8)	61.1 ± 2.2 (−0.1)	69.6 ± 2.2 ^bc^ (1.1)	59.8 ± 2.2 (0.7)
2–3/year	74.5 ± 1.8 ^a^ (1.4)	55.4 ± 1.8 (−2.2)	69.4 ± 1.3 ^ab^ (1.1)	58.2 ± 1.3 (0.2)	69.5 ± 1.1 ^b^ (1.6)	62.5 ± 1.1 (1.4)	70.3 ± 1.2^bc^ (1.8)	59.4 ± 1.2 (0.3)
Never eat	73.2 ± 1.8 ^bc^ (0)	57.9 ± 1.8 (0.3)	69.0 ± 1.3 ^ab^ (0.8)	59.1 ± 1.3 (1.1)	66.8± 1.2 ^acd^ (−1.2)	60.8 ± 1.2 (−0.4)	68.5 ± 1.2^c^ (−0.1)	59.0 ± 1.2 (−0.1)

^a, b, c, d^ Values within a column and country with different superscript letters, differ significantly at *P* < 0.05.
